# The negative impact of xenophobia on compassion with suffering out-group members is attenuated by trait empathy

**DOI:** 10.1038/s41598-022-23776-8

**Published:** 2022-11-08

**Authors:** Thomas Plieger, Sophie Marx, Elena von Gagern, Stefan Bode, Martin Reuter

**Affiliations:** 1 Department of Psychology, University of Bonn, Kaiser-Karl-Ring 9, 53113 Bonn, Germany; 2 Melbourne School of Psychological Sciences, The University of Melbourne, Melbourne, Australia

**Keywords:** Human behaviour, Psychology

## Abstract

Empathy enables human beings to understand and share the internal states of others. Studies show that empathy for pain is higher for in-group compared to out-group members. This might be driven by attitudes and biases towards out-groups. In a between subject design, N = 621 participants filled in questionnaires measuring xenophobia and trait empathy and were presented with photos of suffering individuals either from the in-group or an out-group, which had to be rated with respect to negative affect and the willingness to help the depicted persons. Results do not show more compassion with members of the in-group in general, but a negative effect of xenophobia on state empathy in the out-group condition. Additional moderation analyses show that this effect is less evident in presence of high trait empathy scores. Our results highlight the importance of empathy trainings to attenuate the effects of xenophobic attitudes on social cohabitation in our increasingly polarized and culturally diverse societies.

## Introduction

Empathy is a crucial social cognition and refers to the ability to share the affect of others that helps establishing and maintaining social relationships between both individuals and groups. It enables and motivates human beings to understand and share the internal states of others, thereby promoting the understanding between different individuals and groups. The last decade was characterized by intense worldwide migration flows and an increasing polarization of (at least) western societies. According to a United Nations report in 2016, the worldwide number of migrants in 2015 was 244 million, including 21 million refugees from war and persecution. This constitutes a 40% increase compared to the year 2000. Particularly Northern America and Europe were the main regions of destination of these huge migration flows^1^. Immigration issues played crucial roles in the 2016 US election campaign, and Trump voters in the 2016 election showed significantly lower support to racial equality and disliked globalization and immigration more strongly than voters who did not vote for Trump. Similarly, several European elections (including the vote on Brexit) have been influenced by the heightened salience of immigration, and aversion to Muslim and African immigrants has been the core topic of right-wing populist parties^2,3^. The recent increase in (low-skilled) migration from Muslim and African Countries has produced a stronger support for right-wing, anti-immigrant parties in several European countries^3^. For example, the number of voters for Germany’s populist right-wing party (AfD; “Alternative für Deutschland”) almost tripled between the beginning of the so-called European refugee-crisis in 2015 and 2018^4^. Hence, societal polarization seems to be particularly driven by immigration issues^5^. Similarly, an increase in low-skilled immigrants increased voting polarization in the US^6^. (^3^ provides an explanatory model for the influence of globalization on a stronger political polarization with respect to populism and racial attitudes.) In light of these recent developments, the importance of empathy as an antidote against out-group stereotypes has become particularly evident. As tensions between different groups seem to grow, being able to be empathic towards people who are believed to be different is increasingly important.

Research has produced many different definitions of empathy and, to date, an end of the debate of how empathy really can be defined is not in sight^7,8^. However, most concepts of empathy contain two main dimensions: affective empathy and cognitive empathy. In short, affective empathy is characterized by an affective state that is elicited by the perceived or imagined affective state of another person and that is oriented toward that other person. Some definitions also propose that the affective state has to be similar to the state of the other person (“isomorphism”). Cognitive empathy represents the ability to cognitively and consciously understand the feelings of another person without having to actually feel what the other person feels. Hence, it is mainly operationalized as perspective taking and closely connected to theory of mind (for an in-depth description of the two forms see^8^).

On the one hand, being able to take the perspective of another person and to be emotionally affected by another person’s feelings strengthens the feelings of belongingness and in-group coherence. On the other hand, perceived similarity, e.g., regarding the outer appearance, is an important variable for identifying the belongingness of a person to a certain group and for estimating the genetic kinship to that specific person^9^. It has been shown that empathy toward individuals being perceived as similar and familiar is higher compared to individuals perceived as less similar and less familiar [e.g.,^10–12^. Many studies have shown that empathy for pain is higher when the respective painful situation is observed in an in-group member as compared to an out-group member [e.g.,^10,12–16^. Regarding the cognitive aspects of empathy (i.e. perspective taking), electroencephalography (EEG) activity patterns related to the perception and recognition of emotional faces have been found to be different for in-group vs. out-group members^17^.

The fact that empathy seems to differ dependent on group-membership has become particularly important in the last decade since (western) societies have faced the biggest migration flows in modern history. This has led to pronounced anti-refugee—and particularly to anti-muslim—sentiments, not only in wealthy, western countries, but also in other societies^18–20^. In Europe, refugees are a highly salient group of people because of the massive media coverage, which may have enhanced the subjective perception of immigrants being both real threats (e.g., as potential terrorists) and symbolic threats (e.g., by threatening the values of a society or foreign infiltration). Again, particularly the Islam and people identified as Muslim are often perceived as a threat to liberal western societies and their culture^21,22^. This may lead to more negative feelings and aggression toward this out-group^23,24^. It is conceivable that a higher degree of points of contact can reduce such stereotypes by increasing empathy toward outgroups^25^. Thus, empathy could be a key variable to more pro-social behavior and less hostile attitudes toward out-groups^26^. This seems to be particularly true for perspective taking (i.e. the cognitive component of empathy)^27,28^, although it is unclear whether respective treatments are able to produce long-lasting effects^18^. Contrarily, it can also be assumed that the endorsement of violence against the out-group is enhanced rather than lowered in the presence of high communal concern and empathy for the in-group^29^. Bruneau and colleagues^30^ found that empathy for the in-group negatively predicted prosociality and positively predicted hostile attitudes toward out-groups. Interestingly, parochial empathy (i.e. the difference between empathy for the in-group vs. the out-group) was a better predictor for acceptance of harming members of the out-group than general trait empathy. Thus, empathy felt for a certain in-group may also be an expression of social identity and affectionate belongingness to that group^30^. Consequently, empathy should also be viewed as a group-based emotion and not only an individual interpersonal process^31^.

Taken together, empathy is typically lower for out-groups and may have differential effects on intended prosociality or state empathy depending on group membership of the evaluated individual. State empathy can be understood as an individual’s context-specific empathy in response to a stimulus. In contrast, trait empathy refers to an individual’s general and cross-situational tendency to experience empathy^32,33^. State empathy has been shown to be for important social processes, such as helping others in need, or by being emotionally affected by other persons’ misfortunes^30,34^. Importantly, state empathy is not identical to prosocial behavior or compassion as empathy relates to the affect and cognitions of the agent, whereas prosociality or helping behavior represent the behavioral outcome. Thus, empathy may be seen as a prerequisite for prosocial behavior or compassion. Nevertheless, they can be considered closely related^35–37^, with correlations estimates of more than r = 0.7 between compassion and empathic concern^38^. Consequently, empathy has been operationalized as participants’ emotional response to a person in need of help^36^. Some studies suggest that situational factors such as time pressure^39^ or emotional state^16^ can influence the extent of state empathy. Similarly, individual differences may play a role for the occurrence of state empathy. Several studies showed that empathy for pain or suffering observed in another person is correlated with self-report measures of trait empathy [e.g.,^40–42^]. People with higher scores in trait empathy also have higher empathic accuracy when they rate the pain or negative affect of a suffering person^43^. Therefore, high levels of trait empathy should increase state empathy felt for suffering individuals and readiness for prosocial behavior. In contrast, other individual characteristics may lower empathic states of compassion when observing suffering individuals. In our study, variables that are related to inter-group attitudes are of particular interest. For example, the persistent motivational goal of group-based superiority captured by the concept of social dominance orientation (SDO) and political conservatism have both been shown to be negatively associated with trait empathy [e.g.,^44–46^. In line with this notion, studies found small but significant negative associations between xenophobia and trait empathy^27,45–48^. To the best of our knowledge, there is only little evidence regarding the association of such variables with state empathy. A recent study showed a negative association between SDO and compassion for descriptions of out-group members experiencing something bad or sad^47^. Forgiarini and colleagues^13^ showed that empathy for pain was lowered for suffering out-group members when participants had stronger racial biases in an Implicit Association Test.

Thus, while it has been shown that compassion for out-group members is typically lowered, and trait empathy has been linked to xenophobia, evidence for an association between individual differences in xenophobia and state empathy is scarce so far. Therefore, we wanted to test whether individual levels of xenophobia would be associated with the extent of felt state empathy evoked by visual stimuli in two conditions in which suffering individuals of different groups—a group of Caucasians and a group of alleged Muslims/people from the Middle East—were depicted. Participants were all Germans, a predominantly Caucasian group, which means that the suffering Caucasians were putative in-group members and the suffering Muslims/Middle Easterners were putative out-group members. Furthermore, the aim and the novel aspect of the present study was to investigate whether trait empathy would interact with the association between out-group related xenophobia and state empathy. Specifically, we expected that higher levels of trait empathy could lower the negative association between xenophobia and state empathy (i.e. compassion felt for suffering individuals). As xenophobia is directed toward members of out-groups, we predicted that the moderation effect should only be observed in the out-group condition. Furthermore, we were interested in whether there would be differential effects of cognitive vs. affective trait empathy on state empathy for suffering members of the out-group.

## Methods

### Participants and procedure

Our initial sample comprised N = 665 participants. The vast majority of our sample reported to be born in Germany (n = 631) and to be German citizens (n = 650). To minimize potentially confounding effects regarding whether the presented stimuli would be considered in-group or out-group members, we only included participants who were born in Germany and also were German citizens. We furthermore excluded six participants because of missing data. This resulted in N = 621 participants of whom n = 474 identified as female. N = 145 participants identified as male, and n = 2 identified as non-binary. The mean age was M = 33.73 (SD = 12.70).

The study was conducted online (Unipark, Tivian) and was advertised via social media. Participation was anonymous and all participants provided informed consent after they had received the study information and before proceeding to the study. The study protocol was in accordance with the ethical guidelines of the declaration of Helsinki. The study is part of a broader research project, and all stimuli and self-report questionnaires included were approved by the local ethics committee at the University Hospital of Bonn (No. 014/20). We ran a between-subject design in which participants were randomly assigned to either the in-group or the out-group condition so that all participants saw only one set of photos. After the picture presentation, participants filled in questionnaires measuring trait empathy and xenophobia. All questionnaires and ratings of the photos were presented in German language.

### Self-report measures

Trait Empathy was measured using a German version of the Interpersonal Reactivity Index (IRI)^49^. The actual IRI consists of 4 subscales. However, to save time, and because we had no respective hypotheses, we did not include the fantasy subscale. Consequently, the used version of the IRI comprised 21 items measuring the three subscales perspective taking (Cronbach’s α = 0.76), empathic concern (α = 0.82), and personal distress (α = 0.80), each assessed by 7 items. Although we had no a priori assumptions about the personal distress scale either, we exploratively tested whether self-oriented feelings of personal anxiety and unease when watching people suffer would also interact with xenophobia.

Xenophobia was assessed by two questionnaires, namely the Fear Based Xenophobia scale (FBX)^50^ and the “*Fragebogen zur rechtsextremen Einstellung*” (a German questionnaire measuring right-wing extremist attitudes; FR-LF)^51^. The FBX is a nine item questionnaire that forms a single xenophobia scale with a good internal consistency (α = 0.93). The FR-LF comprises six subscales of which we used five. Anti-semitism was left out to avoid any reactance in our participants, and because we were not interested in this particular aspect of right-wing extremism. The five remaining subscales were *chauvinism*, *hostility to foreigners*, *social Darwinism, trivialization of national socialism*, and *approval of right-winged dictatorship.* The subscales were grouped together into one scale (α = 0.90) for the sake of comprehensibility, as all subscales were substantially correlated with each other (all r > 0.45) and highly correlated with the overall score (all r > 0.72).

Political orientation, as a construct that is related to out-group attitudes [e.g.,^52^, was also assessed by a single item self-rating on a 7-point scale ranging from “left” (1) to “right” (7) on the political spectrum.

### Picture presentation paradigm

To measure state empathy for other people and a possibly resulting prosocial motivation, we presented each participant with a set of 11 photos, depending on their randomly allocated group: (a) either depicting suffering Caucasian people, or (b) suffering people who did not conform to the stereotypic Caucasian appearance. The photos of the latter condition depicted persons with a darker skin type and dark hair so that most Caucasian people would identify the persons as being part of an ethnic out-group. Because we aimed for associations with such strongly undifferentiated and stereotypic category labels, we attempted to cover the most salient and stereotypic aspects in the selected pictures that are typically considered “foreign” in most European societies (e.g., people from Northern Africa and the Middle East). Of note, the participants saw only one of the two picture sets, and the instruction of the picture presentation paradigm did not explicitly refer to group membership, origin, or outer appearance, or used any labels for the depicted persons at all. The two picture sets were further matched such that the same scenarios were shown in both conditions (e.g., an injured man lying on the sideway who is treated by a firefighter, a crying child, or an injured person who is carried by others). In the following, the conditions will simply be referred to as the “in-group” and the “out-group” condition. For the stimulus selection, two of the authors (SM and TP) conducted online picture searches on freely available stock photo databases. We selected the images according to face validity and only used a photo if we found a highly similar one for the other respective experimental condition. More precisely, it was important to find both a person of the in-group and a person of the out-group as the suffering individual in the respective depicted situation (e.g. a crying child or an injured man). To allow readers to assess the pictures and to facilitate replicability, we uploaded all pictures used in our paradigm at https://osf.io/gt3p7/?view_only=ec05920c81b24c4c82a32002ac6cc491. As a manipulation check, each photo was also rated with respect to its valence (“How do you perceive this photo?”; negative to positive) by the participants in our study. As a manipulation check, each photo had to be rated with regard to its valence (“How do you perceive this photo?”; negative to positive).

The second rating dimension of the pictures assessed, to what extent the participants were compassionate and negatively affected by the presented picture (“How much does this photo touch you?”; not at all to very much).

To assess prosocial motivation elicited by the presented photo, we asked our participants to what extent they wanted to alleviate the suffering of the depicted person. Thus, the third rating dimension referred to the helping intention (“How strong is your urge to help the affected person?”; very low to very high).

All three rating dimensions were answered on a 7-point Likert scale.

### Statistical analyses

The core dependent variables were the three rating dimensions for the pictures, for which we first averaged across the individual pictures to obtain mean scores for each dimension. The valence dimension was used as a manipulation check to ensure that the presented photos of the different experimental conditions can be considered as matched. The other two dimensions were used for further analyses to test our hypotheses.

We conducted ANOVAs to test for differences regarding demographic variables and trait empathy in the in-group vs. out-group conditions. The distribution of gender across the conditions was tested using a χ^2^-test. Associations between empathy and xenophobia were tested using Pearson correlations. The analyses were conducted using SPSS 27 (IBM). Moderation effects of xenophobia on the association between trait and state empathy were tested using the SPSS macro PROCESS^53^.

## Results

First, we conducted a manipulation check and compared the two experimental groups in order to rule out any bias due to demographic and trait characteristics. The two groups did not differ in age or educational level. With respect to trait empathy and xenophobia, no differences between the groups were found (see Table 1 for statistical results). Gender was also equally distributed across conditions (χ^2^ = 2.98, df = 2, p = 0.224).

**Table 1:** Mean scores in the samples being presented with photos of in-group versus out-group people in need.

	In-group condition (*N* = 305)	Out-group condition (*N* = 316)	Test of significance
	*M (SD)*	*M (SD)*	
Age	33.76 (12.78)	33.70 (12.65)	F_1,619_ = .003, p = .955, η^2^ < .001
Educational level	4.15 (.99	4.19 (.94)	F_1,619_ = .255, p = .614, η^2^ < .001
FBX	2.38 (1.10)	2.46 (1.02)	F_1,619_ = .821, p = .365, η^2^ = .001
FR-LF	1.47 (.56)	1.50 (.51)	F_1,619_ = .560, p = .455, η^2^ = .001
IRI empathic concern	26.96 (4.93)	27.06 (4.93)	F_1,619_ = .063, p = .802, η^2^ < .001
IRI perspective taking	25.49 (4.69)	25.61 (4.36)	F_1,619_ = .108, p = .743, η^2^ < .001
IRI personal distress	19.79 (5.26)	20.04 (5.18)	F_1,619_ = .331, p = .565, η^2^ = .001
Political orientation	3.10 (1.04)	3.13 (1.01)	F_1,619_ = .070, p = .792, η^2^ < .001
Photo rating: valence	2.87 (.67)	2.79 (.72)	F_1,619_ = 2.197, p = .139, η^2^ = .004
Photo rating: affect	4.59 (.96)	4.56 (.99)	F_1,619_ = .137, p = .711, η^2^ < .001
Photo rating: help	4.48 (1.09)	4.54 (1.11)	F_1,619_ = .593, p = .442, η^2^ = .001

*FBX* fear-based xenophobia, *FR-LF* right wing extremist attitudes, *IRI* interpersonal reactivity index; political orientation (1 = left, 7 = right).

Valence ratings of the photos did not differ significantly between groups, demonstrating that the scenarios were well matched in how negative they were generally perceived to be (Table 1).

Next, we analyzed the other two rating dimensions, negative affect and willingness to help, as our measures of state empathy. The ANOVA showed that there were no main effects of group. Thus, suffering people from the in-group did not evoke more or less negative affect (F_1, 619_ = 0.137, p = 0.711) or willingness to help (F_1, 619_ = 0.593, p = 0.442) than seeing members of the out-group suffer.

Next, we correlated the trait empathy questionnaire results with negative affect and willingness to help. Across conditions, we found significant correlations for both perspective taking (negative affect: r = 0.247, p < 0.001; willingness to help: r = 0.263, p < 0.001) and empathic concern (negative affect: r = 0.492, p < 0.001; willingness to help: r = 0.473, p < 0.001). When we calculated the correlations between trait empathy and the photo ratings (i.e. our measures of state empathy) separately for each condition (in-group and out-group) and tested those for differences, we found that the correlations were not significantly different (all z < 1.3, all p > 0.19) (see Table 2 for detailed correlations in both conditions). The third IRI scale, personal distress, was not at all correlated with negative affect nor willingness to help.

**Table 2:** Correlations between measures of trait empathy and state empathy separated by conditions.

	IRI empathic concern	IRI perspective taking	IRI personal distress	Photo rating: affect	Photo rating: help
	*r* (*p*)				
IRI empathic concern		.431 (< .001)	.215 (< .001)	.451 (< .001)	.436 (< .001)
IRI perspective taking	.430 (< .001)		− .170 (.003)	.212 (< .001)	.224 (< .001)
IRI personal distress	.178 (.001)	− .183 (.001)		.029 (.613)	.025 (.669)
Photo rating: affect	.530 (< .001)	.284 (< .001)	.120 (.033)		.732 (< .001)
Photo rating: help	.509 (< .001)	.303 (< .001)	.124 (.028)	.821 (< .001)	

*IRI* interpersonal reactivity index. Correlations above the diagonal: in-group condition; correlations beneath the diagonal: out-group condition.

In the next step, we correlated trait empathy with xenophobia and the one-item self-rating of political ideology. As there were no differences between experimental groups, correlations were performed for the whole sample. It was found that trait empathy was negatively associated with xenophobia and political conservatism. This held true for cognitive empathy as well as affective empathy. Again, personal distress was not related to political ideology and xenophobia (see Table 3 for all statistics).

**Table 3:** Correlations between measures of trait empathy, xenophobia, and political ideology.

	FBX	FR-LF	Political orientation	IRI EC	IRI PT
	*r* (*p*)				
FR-LF	.762 (< .001)				
Political orientation	.522 (< .001)	.493 (< .001)			
IRI empathic concern	− .182 (< .001)	− .236 (< .001)	− .130 (.001)		
IRI perspective taking	− .203 (< .001)	− .229 (< .001)	− .134 (.001)	.431 (< .001)	
IRI personal distress	*.068 (.090)*	− *.011 (.791)*	− *.058 (.152)*	.197 (< .001)	− .176 (< .001)

*FBX* fear-based xenophobia, *FR-LF* right wing extremist attitudes, *IRI* interpersonal reactivity index; political orientation (1 = left, 7 = right).

For the associations between the photo ratings and xenophobia, we conducted correlations separated by group. Both measures of xenophobia were negatively associated with being negatively affected by the presented photos (FBX: r =  − 0.212, p < 0.001; FR-LF: r =  − 0.243, p < 0.001), and both measures also negatively correlated with willingness to help (FBX: r =  − 0.226, p < 0.001; FR-LF: r =  − 0.213, p < 0.001) in the out-group condition, but not in the in-group condition (where only a negative correlation between FR-LF and affect was found; r =  − 0.154, p = 0.007). In the out-group condition, conservatism was associated with less affect (r =  − 0.177, p = 0.002) and less willingness to help (-0.155, p = 0.006). In the in-group condition, conservatism predicted a stronger willingness to help the suffering people depicted (r = 0.140; p = 0.015) (see Table 4 for all statistics).

**Table 4:** Correlations between state empathy and measures of xenophobia and conservatism in the in-group and out-group condition.

	Photo rating: affect		Photo rating: help	
	In-group	Out-group	In-group	Out-group
	*r* (*p*)			
FBX	− .089 (.122)	− .212 (< .001)	− .015 (.800)	− .226 (< .001)
FR-LF	− .154 (.007)	− .243 (< .001)	− .059 (.302)	− .213 (< .001)
Political Ideology	.077 (.182)	− .177 (.002)	.140 (.015)	− .155 (.006)

*FBX* fear-based xenophobia, *FR-LF* right wing extremist attitudes; political orientation (1 = left, 7 = right).

Finally, we conducted moderation analyses with the two measures of xenophobia as independent variables, the two photo rating dimensions *negative affect* and *willingness to help* as dependent variables and the cognitive and affective aspect of trait empathy as the moderator variables. Thus, we performed 8 moderation analyses for the in-group and the out-group condition with different combinations of our variables in order to test whether trait empathy would moderate the association between xenophobia and the photo ratings. In the in-group condition, there were no moderation effects of trait empathy (i.e., perspective taking or empathic concern measured by the IRI) at all. However, significant moderation effects could be observed in the out-group condition (see Table 5 for all results).

**Table 5:** Moderation analyses in the out-group condition.

X	Y	M	Moderation effect
FBX	Photos: affect	IRI: EC	t = 1.75, p = .080, β = .014
FR-LF	Photos: affect	IRI: EC	t = 1.63, p = .103, β = .023
FBX	Photos: help	IRI: EC	t = 2.08, p = .039, β = .019
FR-LF	Photos: help	IRI: EC	t = 1.68, p = .095, β = .027
FBX	Photos: affect	IRI: PT	t = 1.72, p = .087, β = .019
FR-LF	Photos: affect	IRI: PT	t = 2.21, p = .027, β = .051
FBX	Photos: help	IRI: PT	t = 2.34, p = .019, β = .029
FR-LF	Photos: help	IRI: PT	t = 2.99, p = .003, β = .076

*FBX* fear-based xenophobia, *FR-LF* right wing extremist attitudes, *IRI* interpersonal reactivity index, *PT* perspective taking, *EC* empathic concern.

**Figure 1:**
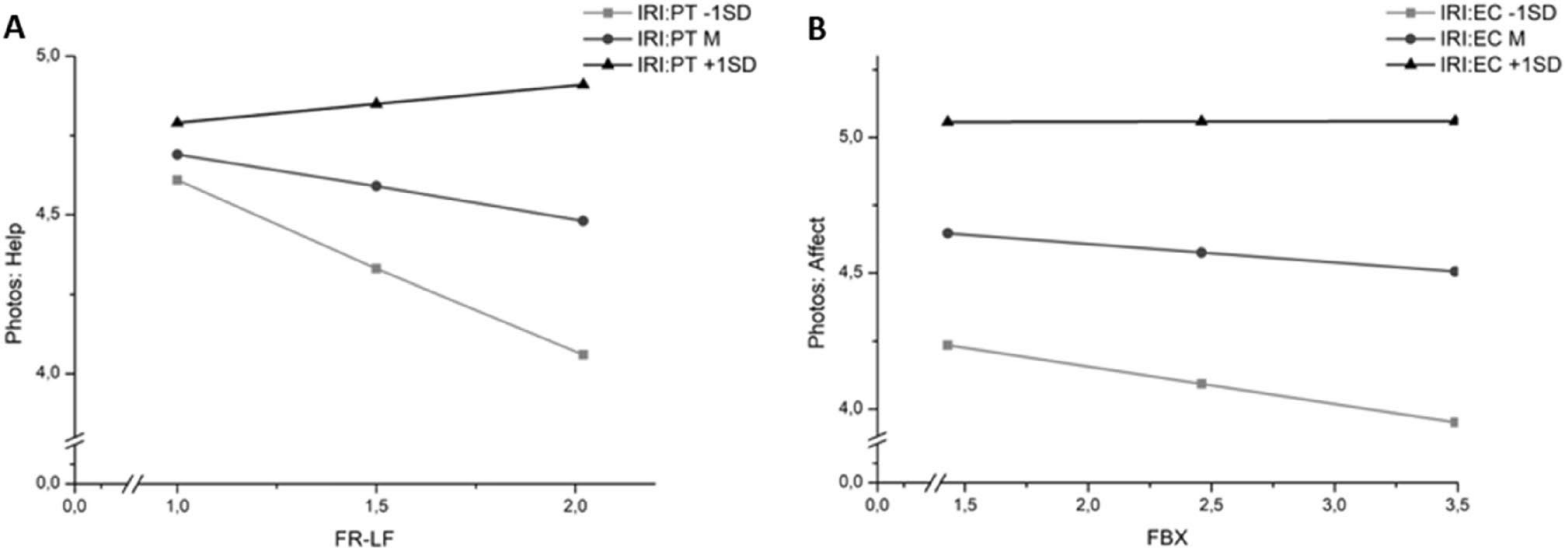
Moderation effects of trait empathy on the association between xenophobia and state empathy. (**A**) strongest moderation effect (p = .003; X = right-wing extremism (FR-LF), M = perspective taking (IRI: PT), Y = willingness to help). (**B**) weakest moderation effect (p = .103; X = fear-based xenophobia (FBX), M = empathic concern (IRI: EC), Y = negative affect in response to photos).

Participants scoring high in trait empathy showed higher state empathy (i.e. negative affect and willingness to help) when observing suffering out-group members irrespective of their xenophobia scores. However, in participants with lower trait empathy scores, there were negative correlations between xenophobia and our measures of state empathy. The moderation effects were somewhat stronger for cognitive empathy and for the photo rating dimension “willingness to help”. Nonetheless, simple slope analyses revealed that the direction of the effect was the same in all moderation analyses although the effects with empathic concern as independent variable were weaker. Figure 1 displays the strongest and the weakest moderation effect. Regarding the strongest moderation effect of perspective taking on the association between willingness to help and right-wing extremism (Fig. 1a), the analysis of conditional effects (i.e. the association between X and Y for different levels of M) showed a highly significant effect (t =  − 3.76, p < 0.001) in participants with low perspective taking scores, whereas the association between right-wing extremism and willingness to help was not significant in participants with medium (t =  − 1.74, p = 0.083) or high (t = 0.64, p = 0.520) scores in perspective taking. Thus, xenophobia was only significantly associated with lower helping intention in individuals with low perspective taking scores. The strongest effect was found for the moderation effect of perspective taking on the association between FR-LF and willingness to help, while the weakest effect was found for the moderation effect of empathic concern on the association between FBX and negative affect. As illustrated in Fig. 1b, the strength of all other moderation effects was somewhere in-between those, but even though not all moderation effects reached significance, the direction of effects was consistent for the out-group.

## Discussion

The first aim of the present study was to investigate whether state empathy, i.e. empathy towards suffering people depicted in different scenarios, would differ depending on whether these people belonged to participants’ racial in-group (here: Caucasian) or to an out-group (here: Muslim/Arabic), and whether there would be differential effects of the participants’ xenophobic attitudes on state empathy. We found that there were no group differences in the respective photo ratings, indicating that there were no differences in state empathy towards members of the in-group vs. the out-group. However, participants’ xenophobia scores measured by the two self-report questionnaires FBX and FR-LF were negatively correlated with the photo rating dimensions “negative affect” and “willingness to help” in the out-group condition, but not the in-group condition. Thus, state empathy with suffering people from the out-group was smaller in participants with more xenophobic attitudes. The second aim and novel aspect of the study was to explore whether trait empathy plays a role in the association between xenophobia and state empathy towards suffering human beings in the in-group vs. the out-group. Indeed, trait empathy moderated the association between xenophobia (measured by the FBX and FR—LF questionnaires) and state empathy (measured by the photo ratings), meaning that the negative correlations were smaller in presence of high levels of trait empathy.

Regarding our first hypothesis that state empathy would be lower toward members of the out-group, there were no main effects of in-group/out-group condition on state empathy; that is, intended helping behavior and the self-reported negative affect were not more pronounced in the in-group condition compared to the out-group condition. This contradicts several studies that found higher empathy for individuals of the respective in-group [e.g.,^12,13,54^. This discrepancy may be due to differences in experimental designs, as many studies used rather artificial stimuli (e.g., faces of different ethnicities for 30 ms or needle pricks in the cheek) [see 15]. In contrast, our stimuli were real-life depictions of suffering individuals that, in turn, are less standardized but arguably more relevant than stimuli used in other studies (e.g., emotional faces). Another explanation could be that our sample generally reported to be quite liberal in terms of its political orientation, even though the mean age suggests that our sample did not mainly comprise students (for which such a trend could be expected). This may likely have counteracted pronounced differences in state empathy between in-group and out-group because people with a liberal political ideology have recently been found to hold fewer negative stereotypes toward ethnic out-groups [e.g.,^55^. Furthermore, it has been shown that liberals tend to be more motivated to show empathy towards both members of the in-group and of out-groups^44^. Thus, a more conservative sample might have produced results overall in line with the mentioned literature that reported more empathy toward in-group members. Our results in relation to personality, which we will discuss in the next paragraph, further support that political orientation is indeed important for understanding these findings.

With respect to individual differences in xenophobic attitudes, state empathy (i.e. the photo ratings of affect and helping intention) was only associated with xenophobia and political conservatism in the out-group condition. This is not surprising because one would expect a certain unwillingness or inability to take the perspective of an out-group in individuals scoring high in xenophobia^56,57^. Consequently, individuals higher on xenophobia are expected to be less compassionate with suffering individuals from an out-group. Similarly, the reported willingness to help was smaller in more xenophobic participants. Although these results may not be surprising, they have important implications for various settings and institutions. Issues with structural racism in the police force, the health care system, and administrative authorities are currently being discussed in many countries [e.g.,^58–62^. For instance, health care workers with racial biases might feel less empathy if a suffering person does not belong to their in-group. In support of this assumption, it has been shown that not only laypeople, but also medical students tended to underestimate the pain of people of color^63^. Whether they are less capable or less motivated to empathize remains a question that needs to be addressed. Several studies suggest that inferior medical treatment of ethnic minorities is also a result of negative attitudes towards out-groups, racial bias, or prejudice^64–66^. Therefore, targeted measures to reduce racial biases and prejudices in health care workers might help reduce structural disadvantages in the health care system^58^. Similar mechanisms are conceivable for other areas that are affected by structural racism.

We also tested for correlational associations between trait empathy and xenophobia. We found significant negative associations between xenophobia and affective as well as cognitive trait empathy. The negative association between xenophobia and trait empathy is in line with results by Nicol and Rounding^45^, although their study did not differentiate between empathic concern and perspective taking. It is conceivable that this association, in turn, is moderated by a third variable. For example, low empathy and xenophobia may both be associated with higher degrees of egocentrism or self-centeredness, which would explain the small, but significant correlations.

Our main finding was that trait empathy moderated the association between xenophobia and state empathy in the out-group condition. Of note, moderation effects could be found in particular for perspective taking, i.e. the cognitive part of trait empathy, whereas there was only a trend in the same direction for empathic concern, which is considered the affective part of empathy. The moderation effects of perspective taking on the association between xenophobia and state empathy suggests that xenophobic attitudes were only relevant for state empathy (for suffering members of an alleged out-group) when cognitive empathy scores were low. Low xenophobic attitudes were associated with higher state empathy and vice versa in participants with less perspective taking. Contrarily, the negative correlations between negative attitudes and willingness to help and xenophobia did not reach significance in participants with higher perspective taking scores. Specifically, participants with higher cognitive trait empathy reported more state empathy with suffering out-group individuals irrespective of their xenophobia scores. Thus, cognitive trait empathy seems to overrule xenophobia when it comes to the question of whether or not to be compassionate and willing to help a suffering member of the out-group. In other words, state empathy toward out-group members does not decrease in individuals with stronger xenophobic attitudes when they have high levels of trait empathy at the same time. This also has important implications for possible interventions that should aim at increasing *trait* empathy. With respect to time-limited situations, a recent study^28^ was able to show that perspective taking was increased after a virtual reality paradigm in which Caucasian participants embodied a person of color. This, in turn, led to less biased behavior in an implicit association task. However, according to Adida et al.^18^ this effect is only short-lived and restricted to behavior, whereas there is no effect on the actual attitude toward an out-group. Our findings of a negative association between perspective taking and xenophobia, whereas the associations with affective empathy are somewhat weaker, are in line with results from a longitudinal study that reported that perspective taking, but not empathic concern, predicted prejudice, whereas prejudice did not predict empathy^27^. Furthermore, the results of our moderation analyses emphasize that it seems to be important to sustainably strengthen cognitive trait empathy (and to a lesser extent also affective trait empathy) in order to increase compassion and willingness to help people irrespective of their group affiliation. This may not entirely prevent xenophobic or racist attitudes, but if trait empathy levels could be increased, this might at least counteract such attitudes in the moment that empathy is required. As a consequence, this could balance out xenophobic tendencies of individuals observing pain or suffering in out-group members, potentially leading to less negatively biased behavioral outcomes. This is particularly, but not exclusively, important in the context of specific institutions in which people are dependent on empathy and help of professionals to receive equal treatment (e.g., the police force, health care workers, or teachers). Several studies suggest that empathy can be significantly improved by training, although it has to be noted that most of the studies investigated health professionals^67,68^. A crucial task for future research will be to extend these findings to other samples. Furthermore, suitable training contexts must be found to reach as many people as possible, if empathy training was to have a broader societally relevant effect. The most obvious option would be to integrate empathy trainings in educational settings such as schooling, although it should be noted that there is the danger accelerating the recent trend to burden teachers with an increasing number of societal and educational tasks^69,70^.

### Limitations and future perspectives

Several limitations to our study must be noted. While our sample size was large and the study well-powered, our sample was not representative with respect to gender distribution and political ideologies as the majority of our participants was female and liberal. However, the mean age of our sample indicated that we did not mainly sample students. Nevertheless, the mean xenophobia scores were also very low and skewed to the lower end of the scale. Despite small variance in our data, we found effects of xenophobia on state empathy. Thus, we would expect even bigger effects in samples with a higher variance in xenophobic attitudes. However, the existence of different relationships at the upper end of the xenophobia scale are also conceivable. We could not test this directly, because the “higher” levels of xenophobia were still relatively low in our sample. It is possible that high levels of perspective taking (i.e. cognitive trait empathy) would not be sufficient to counteract severe xenophobic attitudes and lower their negative effects on state empathy toward out-group members.

With respect to our picture presentation paradigm, we used the valence rating to check that participants perceived the photos of both conditions as equally negative. It would also have been interesting to ask for the perceived severity of the depicted situation, the perceived stress, or other dimensions in which the pictures could have differed. However, the number of potentially relevant dimensions is unlimited, and participants might place a different importance on different dimensions. This highlights the need for more large-scale picture databases in the field (e.g., ^71^) from which researchers could draw pictures that are rated on many dimensions by large samples. However, negative valence was the key dimension in our study, and our participants rated the photos of the in-group and the out-group condition very similar (p = 0.139). The low means of the valence ratings suggest that the photos were strong enough to evoke state empathy (in-group: M = 2.87 vs. out-group: M = 2.79; scale from 1 to 7). Another consideration regarding the stimuli in our picture presentation design is that our out-group condition was quite broad in terms of outer appearance of the persons depicted (e.g. Middle Eastern vs. black African phenotype). It is possible that some pictures evoked more negative feelings than others. However, we did not control for that because the transitions (e.g., in skin color) were fluent and we did not have systematic sub-categories of out-groups.

Similarly, to measure state empathy we only asked for the affect elicited by the respective stimulus and whether the participants were motivated to alleviate the suffering of the depicted person. This way of measuring state empathy is a little bit unspecific because we did not ask for empathy directly. We also could have used more rating dimensions to get a more differentiated measure. For example, Batson et al.^72^ used 20 adjectives to assess empathy toward a protagonist after letting their participants listen to an interview. However, they only presented one interview situation, whereas we presented our participants with eleven photos. For example, using ten rating dimensions instead of two would have led to 110 items instead of 22 in our picture presentation paradigm. This approach might have been more precise, but it would also have led to a higher strain on our participants. Moreover, several other studies measured state empathy by presenting vignettes, emotional expressions, or depictions of people in need of help and asking their participants, how much they were affected by the stimuli or how much they empathized or identified with the protagonists^30,32–34^. Therefore, we believe that our more economical measure of state empathy was sufficient to obtain a valid measurement for how our participants were affected by the photos and how they empathized with the depicted persons in the picture presentation paradigm.

Furthermore, our study is only cross-sectional, meaning that we cannot comment on possible causal associations between (trait) empathy and xenophobia.

Another methodological consideration is that a with-in subject design (as opposed to the between-subject design used here) would allow for testing the very interesting idea of parochial empathy (i.e. the difference between empathy toward members of the in-group and empathy toward members of an out-group in the same people). Bruneau and colleagues found that parochial empathy was an even stronger predictor for tolerating out-group harm than trait empathic concern^30^. In our study, we aimed to avoid exposure to scenarios from both in- and out-group to minimize the risk of stereotypical behavior, and in consequence our participants were either shown suffering people of the in-group *or* from the out-group. This means, we were not able to test for parochial empathy; however, the small positive correlations between conservatism and state empathy in the in-group condition, and the negative association in the out-group condition, nevertheless point in the same direction as the findings of Bruneau et al.^30^. Thus, if conservatism indeed strengthens in-group support and weakens supportive feelings toward out-groups, as the findings of Bruneau and colleagues suggest, this may be explained by the theory of social dominance orientation, which states that people scoring high on SDO aim to maintain existing group hierarchies and imbalances in power and wellbeing. Of note, Bruneau and colleagues asked directly for empathy (i.e., “How much empathy do you feel for the target?”), whereas our assessment was more indirect. Further research in this direction would be very interesting to better understand the association between trait/state empathy and xenophobic behavior and attitudes.

Finally, it must be noted that our picture presentation paradigm does not constitute a behavioral or typically used implicit measure for xenophobia. Our method has the advantage that it is not a classic self-report instrument, which arguably might be prone to self-report bias, but it is also not validated to reveal unconscious or consciously concealed xenophobia. However, typical implicit measures, such as the IAT or the Dotprobe task, are rather artificial and restricted to a laboratory context. One option for future studies to avoid these disadvantages is to measure actual behavioral outcomes. One possible way to do so could be to present participants with NGO donation appeals with depictions of individuals of the out-group and the in-group and to give them the opportunity to donate money (e.g., a certain share of the study compensation).

## Conclusion

The results of our study show that trait empathy and xenophobia interact to moderate the willingness to help suffering people of an outgroup. Trait empathy seemed to overrule xenophobia, meaning that xenophobic attitudes were associated with lower compassion and lower willingness to help out-group members only if trait empathy was low. In people with high trait empathy, xenophobic attitudes became irrelevant for their expressions of compassion and willingness to help when faced with a suffering out-group member.

These findings, while not providing evidence for causal relationships, nevertheless suggest that empathy trainings could be an effective tool for combating the consequences of xenophobic attitudes. Even if effective, empathy trainings will most likely be insufficient to overcome xenophobia, but if they can help to improve state empathy and possibly improve prosocial behavior, this would constitute a significant step towards a better society. Further research is needed to understand how xenophobia can be reduced in the moment when evaluating a crucial situation, and, even more importantly, in the long term.

## Data Availability

All data is publicly available at https://osf.io/gt3p7/?view_only=ec05920c81b24c4c82a32002ac6cc491.

## References

[1] United Nations (2016). *244 million international migrants living abroad worldwide, new UN statistics reveal*. UN Department of Public Information, January 12. Accessed September 2022. https://www.un.org/sustainabledevelopment/blog/2016/01/244-million-international-migrants-living-abroad-worldwide-new-un-statistics-reveal/

[2] Adamson FB, Tsourapas G (2019). Migration diplomacy in world politics. Int. Stud. Perspect..

[3] Rodrik D (2021). Why does globalization fuel populism? Economics, culture, and the rise of right-wing populism. Annu. Rev. Econ..

[4] Schaub M, Gereke J, Baldassarri D (2021). Strangers in hostile lands: exposure to refugees and right-wing support in Germany’s eastern regions. Comp. Pol. Stud..

[5] Silva BC (2018). Populist radical right parties and mass polarization in the Netherlands. Eur. Polit. Sci. Rev..

[6] Mayda AM, Peri G, Steingress W (2022). The political impact of immigration: Evidence from the United States. Am. Econ. J. Appl. Econ..

[7] Cuff BM, Brown SJ, Taylor L, Howat DJ (2016). Empathy: A review of the concept. Emot. Rev..

[8] Walter H (2012). Social cognitive neuroscience of empathy: Concepts, circuits, and genes. Emot. Rev..

[9] Vogt, M. T., Fritzsche, E., & Meißelbach, C. 2016. *Ankommen in der deutschen Lebenswelt: Migranten-Enkulturation und regionale Resilienz in der Einen Welt.* BWV Verlag.

[10] Chiao JY, Mathur VA (2010). Intergroup empathy: how does race affect empathic neural responses?. Curr. Biol..

[11] Miralles A, Raymond M, Lecointre G (2019). Empathy and compassion toward other species decrease with evolutionary divergence time. Sci. Rep..

[12] Xu X, Zuo X, Wang X, Han S (2009). Do you feel my pain? Racial group membership modulates empathic neural responses. J. Neurosci..

[13] Forgiarini M, Gallucci M, Maravita A (2011). Racism and the empathy for pain on our skin. Front. Psychol..

[14] Hoogland CE, Schurtz DR, Cooper CM, Combs DJ, Brown EG, Smith RH (2015). The joy of pain and the pain of joy: In-group identification predicts schadenfreude and gluckschmerz following rival groups’ fortunes. Motiv. Emot..

[15] Molenberghs P, Louis WR (2018). Insights from fMRI studies into ingroup bias. Front. Psychol..

[16] Richins MT, Barreto M, Karl A, Lawrence N (2019). Incidental fear reduces empathy for an out-group’s pain. Emotion.

[17] Gutsell JN, Inzlicht M (2012). Intergroup differences in the sharing of emotive states: Neural evidence of an empathy gap. Soc. Cognit. Aff. Neurosci..

[18] Adida CL, Lo A, Platas MR (2018). Perspective taking can promote short-term inclusionary behavior toward Syrian refugees. Proc. Natl. Acad. Sci..

[19] Lischer SK (2017). The global refugee crisis: Regional destabilization & humanitarian protection. Daedalus.

[20] Narkowicz K (2018). ‘Refugees not welcome here’: State, church and civil society responses to the refugee crisis in Poland. Int. J. Polit. Cult. Soc..

[21] Ciftci S (2012). Islamophobia and threat perceptions: Explaining anti-Muslim sentiment in the West. J. Muslim Minority Affairs.

[22] Velasco González K, Verkuyten M, Weesie J, Poppe E (2008). Prejudice towards Muslims in the Netherlands: Testing integrated threat theory. Br. J. Soc. Psychol..

[23] Domínguez DJF, van Nunspeet F, Gupta A, Eres R, Louis WR, Decety J, Molenberghs P (2018). Lateral orbitofrontal cortex activity is modulated by group membership in situations of justified and unjustified violence. Soc. Neurosci..

[24] Unkelbach C, Forgas JP, Denson TF (2008). The turban effect: The influence of Muslim headgear and induced affect on aggressive responses in the shooter bias paradigm. J. Exp. Soc. Psychol..

[25] Pagotto L, Voci A, Maculan V (2010). The effectiveness of intergroup contact at work: Mediators and moderators of hospital workers' prejudice towards immigrants. J. Community Appl. Soc. Psychol..

[26] Vescio TK, Sechrist GB, Paolucci MP (2003). Perspective taking and prejudice reduction: The mediational role of empathy arousal and situational attributions. Eur. J. Soc. Psychol..

[27] Miklikowska M (2018). Empathy trumps prejudice: The longitudinal relation between empathy and anti-immigrant attitudes in adolescence. Dev. Psychol..

[28] Patané I, Lelgouarch A, Banakou D, Verdelet G, Desoche C, Koun E, Farnè A (2020). Exploring the effect of cooperation in reducing implicit racial bias and its relationship with dispositional empathy and political attitudes. Front. Psychol..

[29] Argo N (2009). Why fight? Examining self-interested versus communally-oriented motivations in Palestinian resistance and rebellion. Secur. Stud..

[30] Bruneau EG, Cikara M, Saxe R (2017). Parochial empathy predicts reduced altruism and the endorsement of passive harm. Soc. Psychol. Pers. Sci..

[31] Smith ER, Mackie DM (2016). Group-level emotions. Curr. Opin. Psychol..

[32] Song Y, Nie T, Shi W, Zhao X, Yang Y (2019). Empathy impairment in individuals with autism spectrum conditions from a multidimensional perspective: A meta-analysis. Front. Psychol..

[33] Zhao Q, Neumann DL, Yan C, Djekic S, Shum DH (2021). Culture, sex, and group-bias in trait and state empathy. Front. Psychol..

[34] Westman M, Shadach E, Keinan G (2013). The crossover of positive and negative emotions: The role of state empathy. Int. J. Stress. Manag..

[35] Decety J, Jackson PL (2004). The functional architecture of human empathy. Behav. Cogn. Neurosci. Rev..

[36] Welp LR, Brown CM (2014). Self-compassion, empathy, and helping intentions. J. Posit. Psychol..

[37] Stocks EL, Lishner DA, Decker SK (2009). Altruism or psychological escape: Why does empathy promote prosocial behavior?. Eur. J. Soc. Psychol..

[38] Pommier E, Neff KD, Tóth-Király I (2020). The development and validation of the Compassion Scale. Assessment.

[39] Gamberini L, Chittaro L, Spagnolli A, Carlesso C (2015). Psychological response to an emergency in virtual reality: Effects of victim ethnicity and emergency type on helping behavior and navigation. Comput. Hum. Behav..

[40] Neumann DL, Westbury HR (2011). The psychophysiological measurement of empathy. Psychol. Empathy.

[41] Singer T, Seymour B, O’doherty J, Kaube H, Dolan RJ, Frith CD (2004). Empathy for pain involves the affective but not sensory components of pain. Science.

[42] Westbury HR, Neumann DL (2008). Empathy-related responses to moving film stimuli depicting human and non-human animal targets in negative circumstances. Biol. Psychol..

[43] Goldstein P, Weissman-Fogel I, Shamay-Tsoory SG (2017). The role of touch in regulating inter-partner physiological coupling during empathy for pain. Sci. Rep..

[44] Hasson Y, Tamir M, Brahms KS, Cohrs JC, Halperin E (2018). Are liberals and conservatives equally motivated to feel empathy toward others?. Pers. Soc. Psychol. Bull..

[45] Nicol AA, Rounding K (2013). Alienation and empathy as mediators of the relation between Social Dominance Orientation, Right-Wing Authoritarianism and expressions of racism and sexism. Pers. Individ. Differ..

[46] Sidanius J, Kteily N, Sheehy-Skeffington J, Ho AK, Sibley C, Duriez B (2013). You're inferior and not worth our concern: The interface between empathy and social dominance orientation. J. Pers..

[47] Hudson SKTJ, Cikara M, Sidanius J (2019). Preference for hierarchy is associated with reduced empathy and increased counter-empathy towards others, especially out-group targets. J. Exp. Soc. Psychol..

[48] Sparkman DJ, Eidelman S, Till DF (2019). Ingroup and outgroup interconnectedness predict and promote political ideology through empathy. Group Process. Intergroup Relat..

[49] Davis MH (1983). Measuring individual differences in empathy: Evidence for a multidimensional approach. J. Pers. Soc. Psychol..

[50] Van der Veer K, Ommundsen R, Yakushko O, Higler L, Woelders S, Hagen KA (2013). Psychometrically and qualitatively validating a cross-national cumulative measure of fear-based xenophobia. Qual. Quant..

[51] Decker O, Hinz A, Geißler N, Brähler E (2013). Fragebogen zur rechtsextremen Einstellung-Leipziger Form (FR-LF). Rechtsextremismus der Mitte. Eine sozialpsychologische Gegenwartsdiagnose.

[52] Duckitt, J. A dual-process cognitive-motivational theory of ideology and prejudice. In *Advances in Experimental Social Psychology* (Vol. 33, pp. 41–113) (Academic Press, 2001).

[53] Hayes, A. F. The PROCESS Macro for SPSS and SAS version 3.0 [Computer software] (2018). Retrieved from afhayes.com.

[54] Stürmer S, Snyder M, Kropp A, Siem B (2006). Empathy-motivated helping: The moderating role of group membership. Pers. Soc. Psychol. Bull..

[55] Beyer S (2020). Relation between college students’ conservatism and negative stereotypes about social groups. Soc. Sci..

[56] Weisz E, Zaki J (2018). Motivated empathy: A social neuroscience perspective. Curr. Opin. Psychol..

[57] Zaki J (2014). Empathy: A motivated account. Psychol. Bull..

[58] Bailey ZD, Krieger N, Agénor M, Graves J, Linos N, Bassett MT (2017). Structural racism and health inequities in the USA: Evidence and interventions. The Lancet.

[59] Johnson TJ (2020). Intersection of bias, structural racism, and social determinants with health care inequities. Pediatrics.

[60] Krieger N (2020). Enough: COVID-19, structural racism, police brutality, plutocracy, climate change—and time for health justice, democratic governance, and an equitable, sustainable future. Am. J. Public Health.

[61] Nordberg A, Twis MK, Stevens MA, Hatcher SS (2018). Precarity and structural racism in Black youth encounters with police. Child Adolesc. Soc. Work J..

[62] Wa Baile, M., Dankwa, S. O., Naguib, T., Purtschert, P., & Schilliger, S. (2019). *Racial Profiling: Struktureller Rassismus und antirassistischer Widerstand* (p. 336). transcript Verlag.

[63] Hoffman KM, Trawalter S, Axt JR, Oliver MN (2016). Racial bias in pain assessment and treatment recommendations, and false beliefs about biological differences between blacks and whites. Proc. Natl. Acad. Sci..

[64] Bailey ZD, Feldman JM, Bassett MT (2021). How structural racism works—racist policies as a root cause of US racial health inequities. N. Engl. J. Med..

[65] Puumala SE, Burgess KM, Kharbanda AB, Zook HG, Castille DM, Pickner WJ, Payne NR (2016). The role of bias by emergency department providers in care for American Indian children. Med. Care.

[66] Nelson K, Norris K, Mangione CM (2002). Disparities in the diagnosis and pharmacologic treatment of high serum cholesterol by race and ethnicity: Data from the Third National Health and Nutrition Examination Survey. Arch. Intern. Med..

[67] Teding van Berkhout E, Malouff JM (2016). The efficacy of empathy training: A meta-analysis of randomized controlled trials. J. Couns. Psychol..

[68] Kataoka H, Iwase T, Ogawa H, Mahmood S, Sato M, DeSantis J, Hojat M, Gonnella JS (2019). Can communication skills training improve empathy? A six-year longitudinal study of medical students in Japan. Med. Teach..

[69] Susteck H (1995). Steigende Erwartungen und steigende Arbeitsbelastung. Die Rahmenbedingungen für Erziehung und Unterricht haben sich grundlegend gewandelt. Realschule in Deutschland.

[70] Weston, K., Ott, M., & Rodger, S. Yet one more expectation for teachers. In *Handbook of School-Based Mental Health Promotion* pp. 105–126 (Springer, 2018).

[71] Crone DL (2018). The Socio-Moral Image Database (SMID): A novel stimulus set for the study of social, moral and affective processes. PLoS ONE.

[72] Batson CD, Chang J, Orr R, Rowland J (2002). Empathy, attitudes, and action: Can feeling for a member of a stigmatized group motivate one to help the group?. Pers. Soc. Psychol. Bull..

